# The role of mood and arousal in the effect of background music on attentional state and performance during a sustained attention task

**DOI:** 10.1038/s41598-024-60218-z

**Published:** 2024-04-25

**Authors:** Luca Kiss, Karina J. Linnell

**Affiliations:** Department of Psychology, Goldsmiths University of London, 8 Lewisham Way, New Cross, London, SE14 6NW UK

**Keywords:** Background music, Mind-wandering, Task-focus, External-distraction, Mood, Arousal, Psychology, Human behaviour

## Abstract

Across two online experiments, this study explored the effect of preferred background music on attentional state and performance, as well as on mood and arousal, during a vigilance task. It extended recent laboratory findings—showing an increase in task-focus and decrease in mind-wandering states with music—to environments with more distractions around participants. Participants—people who normally listen to background music during attention-demanding tasks—completed the vigilance task in their homes both with and without their chosen music and reported their attentional state, subjective arousal, and mood valence throughout the task. Experiment 1 compared music to relative silence and Experiment 2 compared music against the backdrop of continuous noise to continuous noise alone. In both experiments, music decreased mind-wandering and increased task-focus. Unlike in previous laboratory studies, in both experiments music also led to faster reaction times while increasing low-arousal external-distraction states. Importantly, mood and arousal increased with music and were shown to mediate its effects on reaction time and for the first time attentional state, both separately and together. Serial mediation effects were mostly confined to models where mood was entered first and arousal second and were consistent with the mood-arousal account of the impact of background music listening.

## Introduction

People often listen to music and do so for many reasons. Listening to music can for example help us to change our mood (e.g.,^1^), it can change our arousal levels to help us either to relax or to become more energised (e.g.,^2^), and it can evoke mental imagery and mind-wandering (see, e.g.,^3^). Where music is listened to in the background—so where a task is performed concurrently with listening to music—some of the impacts of music are the same, such as its effect on mood and arousal, but others are different^4^. Specifically, music has been found to decrease rather than increase mind-wandering states compared to silence when listened to during performance of a boring vigilance task and, at the same time, to increase task-focus states^5^.

Given that mind-wandering has been linked to negative mood^6–12^ and decreased arousal^13^ and that background music has been shown to increase positive mood (e.g.,^6,14^) and arousal (e.g.^15,16^,) and to decrease mind-wandering states^5^, it can be predicted that background music listening shifts the balance of mind-wandering and task-focus states via its effect on mood and arousal. Taking the link between mind-wandering and arousal into account, while people when over-aroused might seek out music to encourage relaxation and mind-wandering, people performing a boring, non-arousing vigilance task might seek out music to increase their arousal levels and feel more energised for performing the task. This arousal increase in-turn can result in less frequent mind-wandering and more frequent task-focus states. In the latter case, even though music listening requires the dedication of some processing resources to the music (depending on levels of musical expertise^17,18^), it might be that the beneficial effects of background music on mood and arousal and thus attentional state outweigh any drain on processing resources imposed by listening to background music, resulting in more task-focus states and potentially improved performance. The present study aimed to explore, for the first time, whether subjectively reported mood and arousal mediate the effect of background music on attentional state—mind-wandering, task-focus, and also external-distraction states^13^—as well as on performance during a simple attention-demanding task.

## Background

While some studies have found a negative effect of background music (on, e.g., writing fluency^19^, reading comprehension^20^, serial and immediate recall^21–23^, and a range of memory processes^24–26^), others have found a positive effect of background music (on, e.g., reading comprehension in adolescents^27^, response latency during driving^28^, spatial processing^29^, learning^30,31^, reasoning^32^, and sustained attention or vigilance, e.g.,^33–35^).

The seemingly contradictory effects of background music may be a consequence of a variety of factors (for reviews, see^36–38^). These factors include various methodological differences such as (i) the complexity and nature of the task^39^ and the environment in which it is being completed^40^, (ii) the parameters linked to the music, including musicological ones (e.g., lyrics^39^, tempo^41^, tonality^29^), the arousal and mood invoked in listeners by the music^42^, and psychological factors (the liking the listener has for the music^43^ and the familiarity the listener has with the music^44^), as well as (iii) differences between individuals such as in their personality and baseline arousal levels^45^.

All of these factors can either induce or involve different levels of arousal and mood, given that the ‘arousingness’ of, and mood induced by, both tasks and music can vary, as can individual levels of baseline arousal and mood. As regards arousal, there is a non-linear, inverted-U shaped relationship between arousal and performance: only an intermediate arousal level is linked to maximum performance and when arousal is either sub-optimal (i.e., lower than optimal) or supra-optimal (i.e., higher than optimal), performance is impaired^46^ and attentional lapses or off-task attentional states can occur^13^. These attentional lapses can take the form of internally oriented mind-wandering states (i.e., the experience of task-unrelated thoughts) or externally oriented external-distraction states (i.e., the experience of being distracted by environmental stimuli/bodily sensations)^13,47^, where both these states can either be linked to arousal levels that are lower or higher than optimal^47,48^. Moreover, as regards mood, the off-task state of mind-wandering has been linked to negative mood (e.g.,^6–12^.

The experience of attentional lapses is common in contexts where sustaining attention is difficult because tasks are monotonous and boring^49,50^. Given that simple monotonous tasks such as sustained attention or vigilance tasks tend not just to be linked to negative mood (e.g.,^51^) but also to lack of arousal (e.g.,^52,53^), when people are performing these tasks they can be expected to be on the lower side of the inverted-U arousal curve. Then, when they perform the task with background music that has been shown to increase arousal (e.g.,^15,16^, the music might either increase arousal to an intermediate level optimal for task-focused attention and performance on the vigilance task, or it might induce too small an effect such that arousal remains sub-optimal or too large an effect such that arousal becomes supra-optimal. On the other hand, when people are at a supra-optimal arousal level already without music—such as in stressful situations^54^—listening to music while not concurrently performing a task might decrease arousal and shift it to a more optimal intermediate level. In the context of background music listening during performance of a concurrent task, however, although some people indeed report using background music to decrease rather than increase their arousal^4^, there is no study to-date showing an arousal-reducing effect of *background* music. Therefore, in the present study on background music we focused on the potential of music to increase arousal.

In-line with the idea of background music increasing arousal to a more optimal level and consequently increasing task-focused attention or task-focus states, a recent laboratory study by Kiss and Linnell^5^ explored the effect of self-selected or preferred background music on attentional state (including mind-wandering, task-focus, and external-distraction states) and reaction time performance on the Psychomotor Vigilance Task used to measure sustained attention. This laboratory study^5^ found that preferred background music indeed increased task-focus states and did so by decreasing the internally oriented off-task state of mind-wandering whilst not affecting the externally oriented off-task state of external-distraction. However, it did not find an effect of music on reaction time which is in-line with other studies showing no effect of background music on performance measures (including, e.g., reading comprehension^55^, recall^56^, inhibitory functions^57^, and driving accuracy^28^). Nonetheless, an online replication and extension of this laboratory study by Homann, Drody, and Smilek^33^ found that music increased performance on a perceptual decision task with Gabor patches. Regardless of whether there are significant effects of background music on performance, however, many people still choose to listen to it and report doing so during simple tasks both to feel better/increase their mood and feel more energised/increase their arousal^4^.

## Present study

Exploring whether mood and arousal are linked to the effect of background music on attentional state and performance, in the present study we aimed to test whether any effect of music on attentional state and reaction time performance is mediated by mood and arousal, both separately and together. This is relevant to the *mood-and-arousal hypothesis* which highlights that music listened to before a task increases task-performance by increasing the positive mood and arousal of listeners^58–60^. The present study tested, for the first time, the applicability of the mood-and-arousal hypothesis to *attentional state* and examined whether mood and arousal mediate the effect of *background* music on attentional state, in addition to on reaction-time performance. To do so, we extended Kiss and Linnell^5^ by providing an explicit test of the role of both mood—or mood valence as termed here—and arousal, indexed using self-reports elicited throughout the task.

Together with testing the mediation effect of mood and arousal, we also aimed to replicate past findings related to mood and arousal. Regarding mood, we tested whether music is related to mood valence and specifically increases it (e.g.,^6,14^). We also tested whether mood valence is related to attentional state, specifically whether mind-wandering states are linked to decreased mood valence^6–12^ compared to task-focus or external-distraction states. Regarding arousal, in addition to testing the effect of music on arousal, based on findings by Unsworth and Robison^13^ using objective measures of arousal, we expected task-focus states to be linked to an intermediate arousal level and mind-wandering states to be linked to a sub-optimal arousal level. Regarding external-distraction states and arousal levels, there were no strong predictions given that the two pupillometric studies focusing on this topic found opposing results, with external-distraction states being linked to supra-optimal arousal in Unsworth and Robison^13^ and to sub-optimal arousal in Kiss, Szikora, and Linnell^61^.

To complement the focus on mood and arousal, we aimed to generalise past laboratory findings on the effect of music to decrease mind-wandering states and increase task-focus states^5^ to a more ecologically valid context outside the laboratory where distractions might be more likely (for reviews on ecological validity and music, see e.g.,^62,63^). Specifically, the fact that the laboratory study of Kiss and Linnell^5^ did not show any effect of background music on external-distraction states might have been an artefact of conducting the study in an environment that was artificially protected from external distractions^64^. Indeed, in the laboratory study^5^, very few external-distraction states were expressed either with or without music. To allow for the possibility of external-distraction states being experienced, the present online study was conducted in participants’ homes and included different levels of distractions: across two experiments, we explored whether participants report a different pattern of attentional state changes with music in the presence of these different degrees of distractions than in the controlled environment of the laboratory. While Experiment 1 included relative silence as the baseline condition with presumably relatively infrequent distractions from participants’ home environments, Experiment 2 incorporated continuous distractions/noise as the baseline condition.

Although there are some existing studies comparing the effect of background music to noise (e.g.,^65–70^) as in Experiment 2, only very few focused on simple attention tasks and the ones that did showed opposing results: while Hartley and Williams^71^ found that music is more detrimental to performance than white noise on a sustained attention task, Nadon, Tillmann, Saj, and Gosselin^72^ found that noise (in this case, the spectral envelope of each music stimulus applied to a synthesised white noise) is more detrimental to performance than music on a selective-attention task, and Cassidy and MacDonald^25^ showed that on a driving task participants completing the task with self-selected background music performed as accurately with their chosen music as those who completed the task in the presence of car sounds alone (for a review on music and noise during driving, see also^73^).

In sum, we conducted two experiments in each of which participants performed a simple sustained attention task in their homes with and without background music. As already outlined, the main difference between the two experiments was that while in Experiment 1 background music was compared to relative silence, in Experiment 2 continuous background noise (experimentally controlled office noise) was introduced as the baseline condition and in the background music experimental condition music was superimposed on top of this continuous noise. Across the two experiments, we explored, for the first time, whether mood and arousal mediate the effect of music on attentional state. In the mediation analyses, we included attentional states that were affected by background music: if, like in the laboratory, there were to be a decrease in mind-wandering and increase in task-focus states with no effect of music on external-distraction states^5^, then we expected to include in the mediation analysis only these two states, specifically the balance between mind-wandering versus task-focus states. If, however, there were also to be an effect of music on external-distraction states then we expected to conduct on additional mediation analysis on external-distraction states. In addition to attentional state, we included a mediation analysis on reaction time performance. Given that the literature is ambiguous as regards to the order of the causal link between mood valence and arousal (for reviews, see, e.g.,^1,74^ for the effect of music on mood and arousal, and^75^ for the link between mood and arousal), mediation analyses incorporating both orders—mood valence as mediator one and arousal as mediator two, and arousal as mediator one and mood valence as mediator two—were explored.

## Method

### Design

The study had a within subject design; all participants completed the music-present and music-absent conditions in counterbalanced order (i.e., 51 participants completed first the music-present condition and then the music-absent condition, and 55 participants completed first the music-absent condition and then the music-present condition). The main independent variable was music present/absent (music P/A) with relative silence in the presence/absence of music in Experiment 1, and noise in the presence/absence of music in Experiment 2. We also controlled for *time-on-task* (trials from 1 to 170, presented across 5 blocks each containing 34 trials), and *presentation order* (music-present followed by music-absent condition, or music-absent followed by music-present condition). The dependent variables were *thought-probe response* (mind-wandering, task-focus, external-distraction, and mind-blanking states, where mind-blanking states are proposed by Unsworth and Robison^13^ to be a purely low arousal state with arousal levels even lower than those in the other off-task states of mind-wandering and external-distraction), *reaction time* (RT, in milliseconds; ms), *false alarm, mood valence* (measured on a scale from 1 = Very negative, through to 5 = Very positive), and self-rated or *subjective arousal* (measured on a scale from 1 = Not alert at all, through to 5 = Very alert).

The design for the two experiments were the same.

### Participants

#### Experiment 1

Participants were recruited through Goldsmiths University of London’s Psychology Research Participation Scheme and participated in exchange for participation credits. The inclusion criteria were that participants should normally listen to background music during attention-demanding tasks (given that people have been found to perform better in their preferred listening condition^15,76^ and either have a Spotify Prime account or be able and willing to sign up for the free trial version. The latter inclusion criterium was included because participants were asked to create their own music playlist on Spotify so that we could analyse the music tracks after data collection using Spotify’s API endpoints (see details of the music playlists under Stimuli—Preferred background music section).

In total, 106 participants completed the experiment, including 83 females and 22 males (and one participant who did not disclose their sex) between the ages of 18 and 37 (*M* = 20, *SD* = 3.66), all of whom were BSc Psychology students at Goldsmiths University of London. Participants were asked if they “had received any previous musical training (voice or instrument) from a music school, private teacher, or other professional setting, and, if so, for how many years”. There were 54 participants who had received previous formal musical training for an average of 2.7 years (*SD* = 1.535) and 52 who had not received any previous formal musical training. As musical training did not show a significant interaction with music P/A when adding it to the main analyses, analyses reported in the Results section do not include musical training.

#### Experiment 2

Different participants took part in Experiment 2 than in Experiment 1 but, like in Experiment 1, in Experiment 2 participants were recruited through Goldsmiths University of London’s Psychology Research Participation Scheme in exchange for participation credits. The only difference in the inclusion criteria was that, in Experiment 2, only those students could take part who normally listen to background music during attention-demanding tasks completed in a *noisy environment*. Similarly to Experiment 1, participants had to either have a Spotify Prime account or be able and willing to sign up for the free trial version.

In total, 77 participants completed the experiment, including 66 females and 11 males, between the ages of 18 and 42 (*M* = 20.487, *SD* = 4.387), all of whom were BSc Psychology students at Goldsmiths University of London. There were 27 participants who had received previous formal musical training for an average of 4.8 years (*SD* = 3.387) and 50 who had not received any previous formal musical training. As musical training did not show a significant interaction with music P/A when adding it to the main analyses, analyses reported in the Results section do not include musical training.

### Stimuli

#### Preferred background music for Experiment 1

Similarly to in Kiss and Linnell^5^, in this study, self-selected or preferred music was used. Using preferred background music increased ecological validity of the study as it allowed participants to choose tracks that they would normally listen to during attention-demanding tasks and control for personal preferences in music listening. As research by Lynar, Cyejic, Schubert, and Vollmer-Conna^2^ found, compared to pre-selected music tracks, preferred music tracks induced the most joy and positive arousal in listeners and other parameters of the music (such as differences in genre, tempo, arousingness, or lyrics) exerted no significant effect. In this experiment, participants reported a high level of liking for their chosen music tracks (*M* = 4.860, *SD* = 0.908, measured on a scale from 1—Not at all, through to 5—Extremely) and high levels of familiarity with their chosen music tracks (*M* = 3.950, *SD* = 0.859, measured on a scale from 1—Not at all, through to 5—Extremely).

Before the experiment, participants were asked to send a 35- to 40-min-long playlist containing their preferred background tracks to the experimenter prior to participation. There were no restrictions on the music, but participants were asked to send a playlist they would normally listen to when performing a task that requires their attention. They were asked to include tracks in the playlist with similar tempo, lyrical likelihood, and from the same genre. Participants could select tracks of any length but together their playlist had to be at least 35 min long.

Musicological data for each track were collected from the database of the digital music streaming service Spotify^77^. The average tempo of the chosen playlists was 116.502 beats per minute (*SD* = 14.094). Data regarding the lyrical content of playlists was measured by the average ‘lyricalness’ of the chosen playlists which referred to the likelihood of tracks containing any vocal content at any given time point (ranging from 0 = minimum likelihood of a track containing any vocal content, through to 1 = maximum likelihood of a track containing any vocal content). Average lyricalness of the playlists was 0.849 (*SD* = 0.722). Analyses of these musical parameters, namely lyricalness and tempo, showed no significant relationship with any of the dependent variables (thought-probe response, subjective arousal, mood valence, RT, or false alarm).

**Figure 1:**
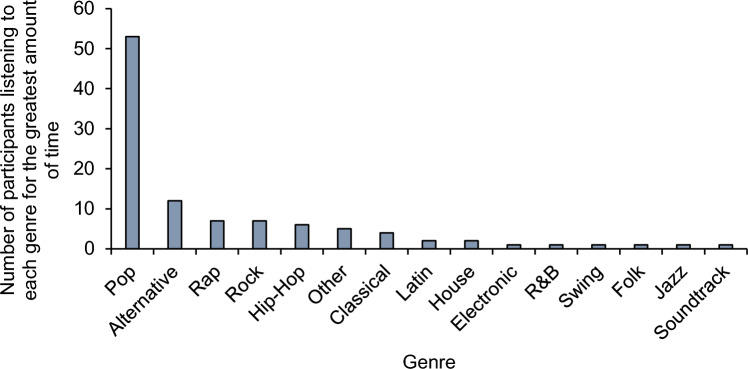
Genres that were most frequently chosen by participants including the number of participants listening to each genre for the greatest amount of time during the music-present part of the experiment. Tracks were placed in the ‘Other’ category if they had any of the following genre-labels: 'apostolic worship', 'sda a cappella', 'kwaito', 'pixel', 'visual kei', 'japanoise', 'afrobeat', 'british dance band', 'canadian singer-songwriter', 'vintage schlager', 'nu gabber', 'gospel', 'christian music', 'adoracao', 'japanese vgm', 'lounge', 'zhongguo feng', 'bossa nova cover', 'nasheed', 'tollywood', 'filmi', 'j-acoustic', 'uk drill', 'celtic', 'middle earth', 'big beat', 'beatboxing', 'chillhop','new tribe', 'chinese minyao', 'ambeat', 'strut', 'stomp and flutter', 'nightrun', 'modern funk', 'australian psych', 'neo-psychedelic', 'brooklyn drill', 'francoton', 'basshall', 'j-pixie'. Playlists from 2 participants could not be downloaded thus data from 104 participants are presented in the figure.

Genres were categorised based on the genre-labels describing the artist and the specific album in which the track appeared; main genre-labels were used to categorise the chosen playlists by using the genres that most frequently occurred in the playlist (see Fig. 1).

#### Preferred background music for Experiment 2

Similarly to in Experiment 1 and in Kiss and Linnell^5^, in Experiment 2, participants’ preferred background music was used. Participants reported a high level of liking for their chosen music tracks (*M* = 4.310, *SD* = 0.853, measured on a scale from 1—Not at all, through to 5—Extremely) and a high level of familiarity with the chosen music tracks (*M* = 4.284, *SD* = 0.952, measured on a scale from 1—Not at all, through to 5—Extremely).

Criteria for creating the music playlist for Experiment 2 were similar to those for Experiment 1 but, for Experiment 2, participants were asked to send a playlist they would normally listen to when performing a task requiring their attention when it was completed *with noise in the background*. 63 of the participants reported that they would use the same playlist without noise in the background, 9 reported that they would use a different playlist, and 2 reported that they do not listen to background music when there is no noise in the background.

Musicological data for each track were collected from Spotify^77^. The average tempo of the chosen playlists was 117.644 beats per minute (*SD* = 28.883) and the average lyricalness of the playlists was 0.829 (*SD* = 0.333). Both the average tempo and lyricalness were similar to in Experiment 1. Analyses of these musical parameters, namely lyricalness and tempo, showed only two significant relationships with the dependent variables: one between music tempo and proportions of mind-wandering states (*p* = 0.004) suggesting that faster tempo music was linked to more mind-wandering states, and the other between music lyrics and proportions of mind-blanking states (*p* = 0.004) suggesting that more instrumental music (music with less lyrics) was linked to more mind-blanking states.

**Figure 2:**
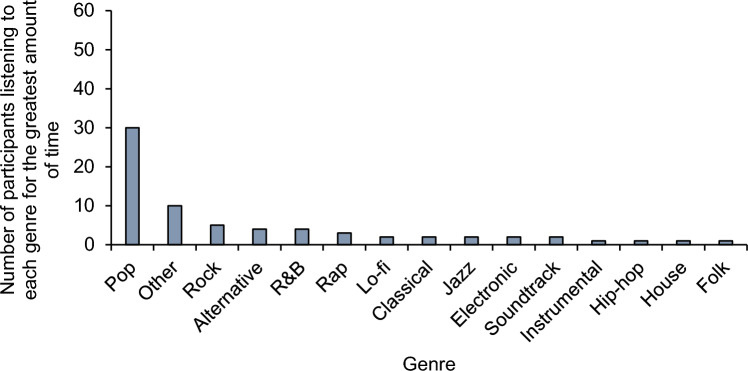
Genres that were most frequently chosen by participants including the number of participants listening to each genre for the greatest amount of time during the music-present part of the experiment. Tracks were placed in the ‘Other’ category if they had any of the following genre-labels: 'apostolic worship', 'sda a cappella', 'kwaito', 'pixel', 'visual kei', 'japanoise', 'afrobeat', 'british dance band', 'canadian singer-songwriter', 'vintage schlager', 'nu gabber', 'gospel', 'christian music', 'adoracao', 'japanese vgm', 'lounge', 'zhongguo feng', 'bossa nova cover', 'nasheed', 'tollywood', 'filmi', 'j-acoustic', 'uk drill', 'celtic', 'middle earth', 'big beat', 'beatboxing', 'chillhop','new tribe', 'chinese minyao', 'ambeat', 'strut', 'stomp and flutter', 'nightrun', 'modern funk', 'australian psych', 'neo-psychedelic', 'brooklyn drill', 'francoton', 'basshall', 'j-pixie'. Playlists from 7 participants could not be downloaded thus data from 70 participants are presented in the figure.

With regard to music genres, Fig. 2 represents the genres that were most frequently chosen by participants.

#### Noise for Experiment 2

The noise played during the task was general office noise without any abrupt changes in frequency and pitch and without any speech (similar noise stimuli were previously used by^65–67, 69–71^). Sources of the noise included sounds of telephones, printers, keyboards, and people (moving around, making general noises such as coughing, or saying made up words but without continuous speech). To generate the noise, the Sound of Colleagues platform (https://soundofcolleagues.com) was used and the noise generated from the platform was then incorporated into the experimental paradigm.

### Materials

#### Psychomotor Vigilance Task

Sustained attention was measured with an online version of the Psychomotor Vigilance Task (containing trials from 1 to 170, presented across 5 blocks each containing 34 trials) developed by Dinges and Powell^78^ which has long been used to measure sustained attention (e.g.,^13^) and has recently been adapted to the context of background music listening^5^. The same online version of the Psychomotor Vigilance Task was completed in both experiments. Successful performance on this task requires maintaining attention on the task, as measured by subjective reports of attentional state and reaction time performance throughout the task. Although the original version of the Psychomotor Vigilance Task only includes ‘go’ trials and only generates a measure of reaction time performance, other well-known sustained attention tasks contain ‘go’ and ‘no-go’ trials and even manipulate their rates to test sustained attention to different extents^79^. To resemble other sustained attention tasks and increase sensitivity, no-go trials were included in the version of the Psychomotor Vigilance Task used here.

On each trial of the task, participants were first presented with a fixation cross in the middle of the screen on a grey background for 2 s (s). Then, they saw a digital clock set to zero (00:00) which, after a variable wait time (equally distributed between 2 and 10 s in 500 ms increments), started counting either upwards or downwards. The task of the participants was to stop the clock counting upwards as soon as they could, by pressing the space bar on their keyboard, but to withhold any response when the clock counted downwards. After pressing the space bar, the clock stopped and remained on the screen for 1 s to provide feedback. Trials when the clock counted downwards were relatively infrequent (presented three times during each block) and responses made to these ‘no-go’ trials were considered as false alarms. For no-go trials, if participants gave a response then the clock stopped counting and the next trial started. If they correctly withheld any response for these no-go trials, then the clock automatically stopped counting at 5 s (but not for go-trials, as for go-trials the clock only stopped after participants made a response) and then the next trial started. No-go trials were presented three times during each of the blocks of 34 trials in a quasi-random distribution with at least five go-trials separating them. This was based on the Sustained Attention to Response Task (SART) developed by Robertson, Manly, Andrade, Baddeley, and Yiend^79^ in which 11% of all trials were no-go trials which translated, in the present case, to 3 no-go trials in each block of 34 trials.

After each trial, a 500-ms blank screen was presented, followed by either the next trial or a probe prompting participants to report their immediately preceding thoughts (to measure their attentional state), hence termed *thought-probes*. Participants completed 5 blocks of 34 trials in each of the music-present and music-absent conditions. One block lasted approximately 5 min. Before starting the experiment, participants completed five practice trials to become familiar with the task.

#### Thought-probes

Similarly to Unsworth and Robison^13^ and Kiss and Linnell^5^, while participants performed the Psychomotor Vigilance Task they were periodically presented with *thought-probes* to measure their attentional state. They were asked to respond to these probes by classifying their immediately preceding thoughts into one of four categorical states similar to the ones used in Unsworth and Robison^13,48^ and in Kiss and Linnell^5^: mind-wandering (statement 1), task-focus (statement 2), external-distraction (statement 3), and mind-blanking (statement 4) states. Mind-blanking states were included to be able to better distinguish between and more accurately measure off-task attentional states^80^. The specific thought-probe question participants were presented with during the task was:

“Please characterise your current conscious experience by choosing one of the following:

1. I am thinking about things unrelated to the task.
2. I am focused on the task or how I am doing it.
3. I am thinking about the things around me (people, sights, sounds, the temperature) or about sensations in my body (hunger, thirst, pain).
4. I am non-alert, or my mind is blank.”

In all, they were presented with six thought-probes per block of 34 trials. The six thought-probes were randomly distributed within each block.

#### Subjective arousal

After each attentional state thought-probe, participants were asked: “indicate how alert you feel at this moment” on a scale from 1 (Not alert at all) through to 5 (Very alert). This question was based on past background music studies using subjective measures of arousal (e.g.,^81^). Nonetheless, this subjective measure of arousal was used as an exploratory measure in the present study given that research on the correlation between subjective arousal and physiological arousal responses to music do not always agree (for a discussion, see^82^): while some studies found a significant correlation between subjective measures and physiological (in this case pupillometric) measures of arousal in relation to music (e.g.,^83^), others reported only a low correlation between physiological (in this case heart rate) and subjective measures of arousal (e.g.,^84^).

#### Mood valence

After each thought-probe and arousal question, participants were asked to “indicate how negative / positive your mood is” on a scale from 1 (Very negative) through to 5 (Very positive). Mood valence in relation to music has often been measured with self-reports^85^ and research has suggested that self-reports of mood valence relating to current experiences can be considered as relevant and valid measures^86^.

### Procedure

#### Experiment 1

Experiment 1 was approved by the Psychology Department Ethics Committee at Goldsmiths University of London on the 10th of November 2020. It was performed in accordance with relevant guidelines and regulations and all participants signed a consent form prior to completing the experiment. Participants were recruited online and completed the study in their homes. After signing up to the experiment and sending a music playlist to the experimenter, they were emailed a link to the Qualtrics survey that contained the information sheet, consent form, GDPR, as well as the main study questions and the link to the experimental paradigm. Participants first completed the questions related to their demographic background (biological sex, education, musical training). Then, they were presented with an embedded link to the experimental paradigm that was created in Psychopy and hosted on the online platform Pavlovia (www.pavlovia.org): they were asked to open the paradigm in a new window while keeping the Qualtrics survey open and return to it once they had finished the experimental task.

After opening the link to the experimental paradigm, participants were presented with the instructions for completing the task. Specifically, they were asked to equip themselves with their musical playlist and a pair of headphones. They were asked to complete the task in a quiet room with minimal distractions around them and to put their phone in ‘do not disturb’ mode. Finally, they were asked to close all other websites (apart from those supporting the survey and the paradigm) on their browser and all other applications to make sure the paradigm could run smoothly. They were then presented with instructions about the nature of the Psychomotor Vigilance Task and were presented with five practice trials. One of the practice trials included a thought-probe, arousal-, and mood-related question.

After the practice trials, they were asked if they understood the task and if they wanted to repeat the practice trials. They were also presented with the instructions about the task again to refresh their memory. When they were ready, they could start the main task. There were five blocks of 34 trials in the music-present and five blocks of 34 trials in the music-absent condition. Between blocks, participants could have an optional three-minute break. Between music-present/music-absent (or music P/A) conditions, participants had a compulsory ten-minute break during which they could either sit back and relax or play a word-search game unrelated to the task (on the website https://api.razzlepuzzles.com/wordsearch which was stated on the screen). The ten-minute break was added to reduce the carry-over effects of the music and help participants recover from the fatigue caused by the first five blocks (e.g.,^87^). Although they were explicitly asked to start playing the music just before the music-present condition and to continuously play it throughout the music-present condition, but stop it for the music-absent condition, whether they followed these instructions was not otherwise checked during the task. Nevertheless, even if they listened to or did not listen to music as instructed, not following the instructions would have only masked any effects of music making it more difficult to find significant effects, rather than making them stronger.

Nevertheless, after completion of both the music-present and music-absent halves of the experimental task, participants returned to the Qualtrics survey to answer some questions about their liking for, and familiarity with, their chosen music playlist. They were also asked to report whether there had been any distractions around them during completion of the task on a scale from 1 (Not at all) through to 7 (All the time) and, if so, what the nature of these distractions was. Overall, participants reported some level of distraction (*M* = 2.472, *SD* = 1.686) and most of the reported distractions were auditory in nature such as people talking within earshot or car noises from the street. Additionally, they were asked whether they had been multitasking while completing the experimental task, on a scale from 1 (Not at all) through to 7 (All the time) and, if so, what they had been doing. Overall, they reported a low level of multitasking (*M* = 1.660, *SD* = 1.059) and most of the reported multitasking consisted of checking their phones, talking, eating, drinking, or singing.

After completion of the Qualtrics survey, participants submitted their responses, indicated their username to receive credits for their participation, and were presented with a short written debrief.

#### Experiment 2

Experiment 2 was approved by the Psychology Department Ethics Committee at Goldsmiths University of London on the 20th of September 2022. It was performed in accordance with relevant guidelines and regulations and all participants signed a consent form prior to completing the experiment. Participants were recruited online and completed the study in their homes. The procedure was the same as in Experiment 1: after signing up to the experiment and sending the music playlist to the experimenter, they were emailed a link to the Qualtrics survey that contained the information sheet, consent form, GDPR, as well as the main study questions and the link to the experimental paradigm. Participants first completed the questions related to their demographic background and were then presented with an embedded link that directed them to the experimental paradigm (the online Psychomotor Vigilance Task also used in Experiment 1) that was created in Psychopy and hosted on the online platform Pavlovia (www.pavlovia.org). They were asked to open the paradigm in a new window while keeping the Qualtrics survey open and return to it once they had finished the experimental task.

Participants were instructed to either avoid using noise cancelling headphones or turn the noise cancelling feature off. They were asked to play the music via their phones and to turn the volume of the music to a level that they would normally listen to. After calibrating the music volume, they could stop listening to the music. Then, they were presented with instructions for calibrating the volume of the noise. At this point, the office noise automatically started playing from their computer speakers and they were asked to ensure that their speakers were working and that they could hear the noise. Then, during the noise calibration itself, they were asked to restart listening to the music via their headphones and to turn the volume of the noise to an audible but not overpowering level which they could hear even when the music was playing. Specifically, they were asked to ensure that they could clearly hear all sources in the noise, including telephones, keyboards, printers, and people. They were told that the volume of the noise should be similar to the volume of noise experienced in an office, café, or library. Once they had calibrated the volume of the noise, they were asked not to alter the volume settings on the computer speaker throughout the experiment. Then, similarly to in Experiment 1, they were asked to complete the task in a quiet room with minimal distractions around them (other than the noise that was part of the task), to put their phones in ‘do not disturb’ mode, and to avoid multitasking while completing the task. They could then start the experimental task that was the same as in Experiment 1.

After completion of the experimental task, participants returned to the Qualtrics survey to answer some questions about their liking for, and familiarity with, their chosen music playlist. Participants were then asked to report whether there had been any distractions around them during completion of the task on a scale from 1 (Not at all) through to 7 (All the time) and, if so, what the nature of these distractions was. Overall, participants reported some level of distraction (*M* = 2.365, *SD* = 1.607) and most of the reported distractions were auditory in nature such as people walking by or talking, the phone ringing, and sounds from the street such as construction noise. Additionally, they were asked whether they had been multitasking during completion of the task on a scale from 1 (Not at all) through to 7 (All the time) and, if so, what they had been doing. Overall, participants reported a low level of multitasking (*M* = 1.636, *SD* = 0.994) and most of the reported multitasking was checking their phones, talking, and eating or drinking.

After completion of the Qualtrics survey, participants submitted their responses, indicated their username to receive credits for participation, and were presented with a short written debrief.

### Ethical approval

All procedures performed in this study involving human participants were in accordance with the 1964 Helsinki declaration and its later amendments or comparable ethical standards, and approved by the institutional research committee at Goldsmiths University of London (Psychology Department Ethics Committee at Goldsmiths, University of London, 10th of November 2020—Experiment 1, and 20th of September 2022—Experiment 2) and with the 1964 Helsinki declaration and its later amendments or comparable ethical standards.

### Informed consent

Informed consent was obtained from all individual participants included in the study.

## Results

The present study aimed to explore whether the effect of preferred background music on attentional state is mediated by mood and arousal. Additionally, it aimed to generalise past laboratory findings to environments with more distractions around participants. The main independent variable was music present/absent (in Experiment 1, relative silence in the presence/absence of music and, in Experiment 2, noise in the presence/absence of music). The other variables were time-on-task (trial number 1–170 presented across 5 blocks containing 34 trials in each block) and presentation order (music-present followed by music-absent condition, or music-absent followed by music-present condition); their effects were controlled for in the analyses by adding time-on-task as a covariate and presentation order as a random effect to the linear mixed models (see below). The dependent variables were thought-probe response (mind-wandering, task-focus, external-distraction, and mind-blanking states), reaction time (RT, in ms), false alarm, mood valence, and subjective arousal.

All analyses were extensions of those conducted by Unsworth and Robison^13^ and Kiss and Linnell^5^. In the present study—in addition to the mediation analyses—linear mixed models were conducted on the data so that, in analyses of thought-probe response, cells that were empty as a result of collecting thought-probe response only after a sub-set of trials could be accounted for. To control for time-on-task effects, time-on-task was added to the models as a covariate when testing the effect of music P/A on the dependent variables. To control for presentation order effects, presentation order was added to the models as a random effect when testing the effect of music P/A on the dependent variables, following Barr’s recommendation to choose the maximum random effect structure^88^.

Before reporting results relevant to the main focus of this study on the mediation effects of mood and arousal together, we report analyses exploring how results of Experiments 1 and 2 relate to past findings. These baseline analyses were important as they helped determine which attentional states are affected by music and thus should be included in the mediation analyses. Therefore, analyses focusing on each dependent variable in turn are first reported, including thought-probe response, RT, false alarm, mood valence, and subjective arousal. These analyses showed that in both experiments music led to faster RTs, decreased mind-wandering states and on the one hand increased task-focus states and on the other hand increased external-distraction states. Therefore, we conducted mediation analyses focused on predicting RT as well as on predicting on the one hand the balance of mind-wandering and task-focus states, and on the other hand the balance of mind-wandering and external-distraction states. Given that mood valence and subjective arousal separately mediated the effect of music P/A (relative silence in the presence/absence of music in Experiment 1, and noise in the presence/absence of music in Experiment 2) on RT and on the balance of these thought-probe responses, serial mediation analyses were performed focusing on the mediation effect of both mood valence and arousal together. In the Results section, simple mediation analyses focusing on mood valence and arousal separately are reported under the subsections dedicated to mood and arousal (entitled ‘Mood valence’ and ‘Subjective arousal’) and then at the end of the Results section serial mediation analyses are reported in which mood valence and arousal were entered together in one model, both with mood entered first and arousal second, and arousal entered first and mood second.

### Thought-probe response

Relevant to the dependent variable thought-probe response, a linear mixed model was conducted exploring the effect of music P/A (relative silence in the presence/absence of music in Experiment 1, and noise in the presence/absence of music in Experiment 2) on thought-probe response for each of the different attentional-state categories separately (mind-wandering, task-focus, external-distraction, and mind-blanking states). For these analyses, the dependent variable thought-probe response was added to each model as a binary dependent variable, so that in one model mind-wandering states were compared to all other states as the reference category, in another task-focus states were compared to all other states as the reference category, in another external-distraction states were compared to all other states as the reference category, and in the last mind-blanking states were compared to all other states as the reference category. In the models, subjects and presentation order were entered as random effects and music P/A as a fixed effect.

#### Experiment 1

**Figure 3:**
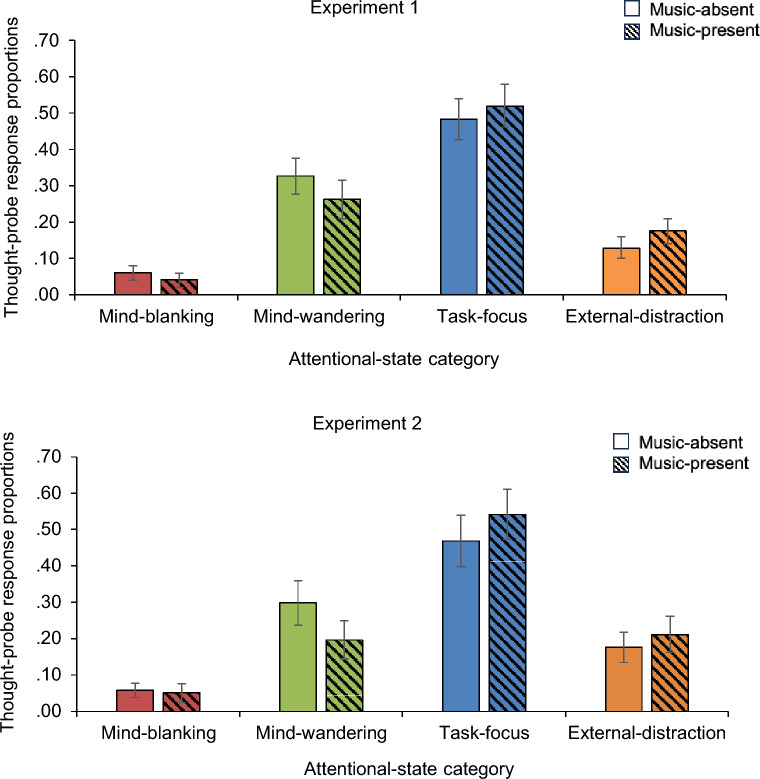
Thought-probe response proportions averaged across presentation order as a function of music-present/absent (i.e., relative silence in the presence/absence of music in Experiment 1, and noise in the presence/absence of music in Experiment 2). Error bars represent 95% confidence intervals.

The linear mixed model exploring the effect of music P/A (relative silence in the presence/absence of music) on task-focus states showed a significant main effect of music P/A, showing that participants reported less task-focus states in the music-absent than in the music-present condition, *t*(6358) = −2.804, *p* = 0.005 (*b* = −0.181, *SE* = 0.064; see Fig. 3 for a summary of the relative proportions of the different thought-probe responses).

The linear mixed model exploring the effect of music P/A (relative silence in the presence/absence of music) on mind-wandering states showed a significant main effect of music P/A, showing that participants reported more mind-wandering states in the music-absent than in the music-present condition, *t*(6358) = 6.634, *p* < 0.001 (*b* = 0.437, *SE* = 0.066).

The linear mixed model exploring the effect of music P/A (relative silence in the presence/absence of music) on external-distraction states showed a significant main effect of music P/A, showing that participants reported less external-distraction states in the music-absent than in the music-present condition, *t*(6358) =  −5.910, *p* < 0.001 (*b* = −0.454, *SE* = 0.077).

The linear mixed model exploring the effect of music P/A (relative silence in the presence/absence of music) on mind-blanking states showed a significant main effect of music P/A, showing that participants reported more mind-blanking states in the music-absent than in the music-present condition, *t*(6358) = 4.574, *p* < 0.001 (*b* = 0.464, *SE* = 0.101).

#### Experiment 2

The linear mixed model exploring the effect of music P/A (noise in the presence/absence of music) on task-focus states showed a significant main effect of music P/A, showing that participants reported less task-focus states in the music-absent than in the music-present condition, *t*(4618) =  −4.898, *p* < 0.001 (*b* = -0.382, *SE* = 0.078; see Fig. 3 for a summary of the relative proportions of the different thought-probe responses).

The linear mixed model exploring the effect of music P/A (noise in the presence/absence of music) on mind-wandering states showed a significant main effect of music P/A, showing that participants reported more mind-wandering states in the music-absent than in the music-present condition, *t*(4618) = 8.709, *p* < 0.001 (*b* = 0.741, *SE* = 0.085).

The linear mixed model exploring the effect of music P/A (noise in the presence/absence of music) on external-distraction states showed a significant main effect of music P/A, showing that participants reported less external-distraction states in the music-absent than in the music-present condition, *t*(4618) = −3.202, *p* = 0.001 (*b* = −0.278, *SE* = 0.087).

The linear mixed model did not show any significant effects of music P/A (noise in the presence/absence of music) on mind-blanking states (*p* = 0.397).

### Performance measures

A linear mixed model was conducted exploring the effect of music P/A (relative silence in the presence/absence of music in Experiment 1, and noise in the presence/absence of music in Experiment 2) on the continuous dependent variable of reaction time (RT). For this linear mixed model, subjects and presentation order were entered as random effects and music P/A as a fixed effect. For analyses on RT, similarly to Kiss and Linnell^5^, numbers below 100 ms or above 5000 ms were excluded (in total 1084 data points) because RT below 100 ms were assumed to be false starts^89^ and RTs above 5000 ms assumed to be outliers (because they were longer than the time required for the analogue version of the clock to complete a full revolution^5^). Excluding RTs below 100 ms as false starts also ensured that RTs could be measured accurately given that, as a recent study highlighted, online platforms are reasonably accurate and reliable for measuring RTs above 100 ms^90^.

The second performance measure was false alarm. A linear mixed model was conducted to explore the effect of music P/A (relative silence in the presence/absence of music in Experiment 1, and noise in the presence/absence of music in Experiment 2) on false alarms. False alarm was a binary dependent variable derived from responses to no-go trials that were either correct withholds or false alarms. In this model, subjects and presentation order were entered as random effects and music P/A as a fixed effect.

Then, a linear mixed model was conducted exploring the relationship between the main objective performance measure, RT, and attentional-state category (by measuring RTs for trials immediately after which participants reported the given attentional-state category). In this model, attentional-state category was entered as a fixed effect and subjects were entered as random effects.

#### Experiment 1

**Figure 4:**
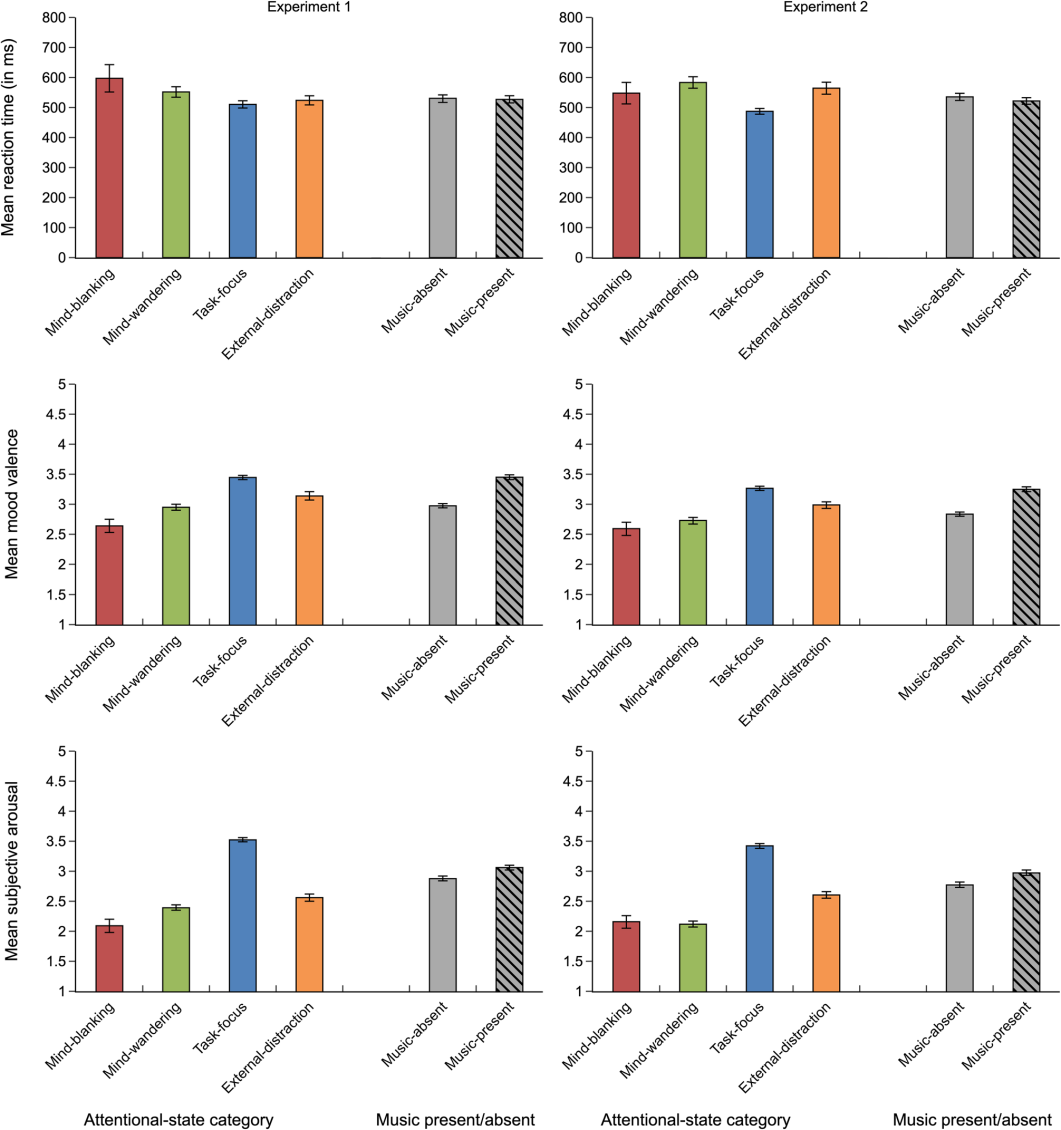
Mean reaction time (in ms), mood valence, and subjective arousal as a function of attentional-state. category and music P/A. Error bars represent 95% confidence intervals.

The linear mixed model exploring the effect of music P/A (relative silence in the presence/absence of music) on RT showed a main effect of music P/A on RT, showing that participants had faster RTs in the music-present than in the music-absent condition, *t*(7531.235) = 1.982, *p* = 0.047 (*b* = 7.785, *SE* = 3.928; see Fig. 4).

The linear mixed model conducted to explore the effect of music P/A (relative silence in the presence/absence of music) on false alarms (no-go trials to which participants reacted versus did not react) showed no significant results (*p* = 0.726).

To explore the relationship between the main performance measure, RT, and attentional-state category, a linear mixed model analysis was performed. The model showed that task-focus states were linked to faster RTs than mind-blanking states, *t*(6301) = 4.084, *p* < 0.001 (*b* = 81.090, *SE* = 19.853) and mind-wandering states, *t*(6301) = 4.863, *p* < 0.001 (*b* = 48.328, *SE* = 9.937; see Fig. 4).

#### Experiment 2

The linear mixed model exploring the effect of music P/A (noise in the presence/absence of music) on RT showed a main effect of music P/A on RT, showing that participants had faster RTs in the music-present than in the music-absent condition, *t*(6109.310) = 2.170, *p* = 0.030 (*b* = 8.100, *SE* = 3.733; see Fig. 4).

The linear mixed model conducted to explore the effect of music P/A (noise in the presence/absence of music) on false alarms (no-go trials to which participants reacted versus did not react) showed a significant main effect of music P/A, showing that false alarms were lower in the music-present (*M* = 0.660, *SD* = 0.452) than in the music-absent condition (*M* = 0.710, *SD* = 0.473), *t*(2306) = −2.911, *p* = 0.004 (*b* = −0.270, *SE* = 0.093).

To explore the relationship between the main performance measure, RT, and attentional-state category, a linear mixed model analysis was performed. The model showed that task-focus states were linked to faster RTs than mind-blanking states, *t*(4180) = 3.243, *p* = 0.001 (*b* = 60.584, *SE* = 18.680), mind-wandering states, *t*(4180) = 9.590, *p* < 0.001 (*b* = 96.147, *SE* = 10.026), and external-distraction states, *t*(4180) = 7.040, *p* < 0.001 (*b* = 77.100, *SE* = 10.951; see Fig. 4).

### Mood valence

A linear mixed model was conducted exploring the effect of music P/A (relative silence in the presence/absence of music in Experiment 1, and noise in the presence/absence of music in Experiment 2) on the continuous dependent variable of mood valence. For this linear mixed model, subjects and presentation order were entered as random effects and music P/A as a fixed effect. Then, a linear mixed model was conducted exploring the relationship between mood valence and attentional-state category (i.e., by including self-reports of mood valence for trials after which participants reported the given attentional-state category). In this model, attentional-state category was entered as a fixed effect and subjects were entered as random effects. Finally, three mediation analyses were performed to explore whether mood valence mediated the effect of music P/A on (i) the balance of mind-wandering versus task-focus states (decrease in mind-wandering and increase in task-focus states), (ii) the balance of mind-wandering versus external-distraction states (decrease in mind-wandering and increase in external-distraction states), and (iii) RT, using the 4.0 version of the PROCESS macro (an observed variable ordinary least squares and logistic regression path analysis modelling tool^91^) with bootstrapping procedures for the indirect effects (unstandardised indirect effects were computed for each of 5000 bootstrapped samples).

#### Experiment 1

A linear mixed model was performed to explore the effect of music P/A (relative silence in the presence/absence of music) on mood valence. The model showed a significant main effect of music P/A, showing that participants reported more positive mood valence in the music-present than in the music-absent condition, *t*(1366.863) = −17.129, *p* < 0.001 (*b* = −0.476, *SE* = 0.028, see Fig. 4).

A linear mixed model was performed to explore the relationship between mood valence and attentional-state category. The linear mixed model showed that task-focus states were linked to significantly more positive mood valence than mind-wandering states, *t*(6356) = −16.569, *p* < 0.001 (*b* = −0.496, *SE* = 0.030), than mind-blanking states, *t*(6356) = −13.507, *p* < 0.001 (*b* = −0.807, *SE* = 0.060) and than external-distraction states, *t*(6356) = −8.181, *p* < 0.001 (*b* = −0.309, *SE* = 0.038; see Fig. 4).

The mediation analyses showed that mood valence significantly mediated the effect of music P/A (relative silence in the presence/absence of music) on the balance of mind-wandering versus task-focus states, *IE* = 0.153, 95% lower limit *CI* = 0.125, 95% upper limit *CI* = 0.184, on the balance of mind-wandering versus external-distraction states, *IE* = 0.064, 95% lower limit *CI* = 0.019, 95% upper limit *CI* = 0.111, and on RT, *IE* = −10.297, 95% lower limit *CI* = −15.445, 95% upper limit *CI* = −5.283.

#### Experiment 2

A linear mixed model was performed to explore the effect of music P/A (noise in the presence/absence of music) on mood valence. The model showed a significant main effect of music P/A, showing that participants reported more positive mood valence in the music-present condition than in the music-absent condition, *t*(916.607) = −13.876, *p* < 0.001 (*b* = −0.435, *SE* = 0.031; see Fig. 4).

A linear mixed model was performed to explore the relationship between mood valence and attentional-state category. The linear mixed model showed that task-focus states were linked to significantly more positive mood valence than mind-blanking states, *t*(4616) = −11.862, *p* < 0.001 (*b* = −0.671, *SE* = 0.057), than mind-wandering states, *t*(4616) = −17.404, *p* < 0.001 (*b* = −0.535, *SE* = 0.031) and than external-distraction states, *t*(4616) = −8.239, *p* < 0.001 (*b* = −0.276, *SE* = 0.034; see Fig. 4).

The mediation analyses showed that mood valence significantly mediated the effect of music P/A (noise in the presence/absence of music) on the balance of mind-wandering versus task-focus states, *IE* = 0.293, 95% lower limit *CI* = 0.238, 95% upper limit *CI* = 0.356, on the balance of mind-wandering versus external-distraction states, *IE* = 0.138, 95% lower limit *CI* = 0.078, 95% upper limit *CI* = 0.203, and also on RT, *IE* = −13.335, 95% lower limit *CI* = −18.362, 95% upper limit *CI* = −8.767.

### Subjective arousal

First, a linear mixed model was conducted exploring the effect of music P/A (relative silence in the presence/absence of music in Experiment 1, and noise in the presence/absence of music in Experiment 2) on the continuous dependent variable of subjective arousal. For this linear mixed model, subjects and presentation order were entered as random effects and music P/A as a fixed effect. Then, a linear mixed model was conducted exploring the relationship between subjective arousal and attentional-state category (by including arousal self-reports for trials after which participants reported the given attentional-state category). In this model, attentional-state category was entered as a fixed effect and subjects were entered as random effects. Finally, three mediation analyses were performed to explore whether subjective arousal mediated the effect of music P/A on (i) the balance of mind-wandering versus task-focus states (decrease in mind-wandering and increase in task-focus states), (ii) the balance of mind-wandering versus external-distraction states (decrease in mind-wandering and increase in external-distraction states), and (iii) RT, using the 4.0 version of the PROCESS macro (an observed variable ordinary least squares and logistic regression path analysis modelling tool^91^) with bootstrapping procedures for the indirect effects (unstandardised indirect effects were computed for each of 5000 bootstrapped samples).

#### Experiment 1

A linear mixed model was conducted to explore the effect of music P/A (relative silence in the presence/absence of music) on subjective arousal. The model showed a significant main effect of music P/A, showing that subjective arousal was higher in the music-present than in the music-absent condition, *t*(1768.765) = −6.615, *p* < 0.001 (*b* = -0.174, *SE* = 0.026; see Fig. 4).

The linear mixed model exploring the relationship between subjective arousal and attentional-state category showed that task-focus states were linked to significantly higher arousal than mind-wandering states, *t*(6356) = −38.681, *p* < 0.001 (*b* = −1.131, *SE* = 0.029), than mind-blanking states, *t*(6356) = −24.529, *p* < 0.001 (*b* = −1.430, *SE* = 0.058) and than external-distraction states, *t*(6356) = −26.108, *p* < 0.001 (*b* = −0.962, *SE* = 0.037; see Fig. 4).

The mediation analyses showed that subjective arousal mediated the effect of music P/A (relative silence in the presence/absence of music) on the balance of mind-wandering versus task-focus states, *IE* = 0.126, 95% lower limit *CI* = 0.087, 95% upper limit *CI* = 0.167, on the balance of mind-wandering versus external-distraction states, *IE* = 0.018, 95% lower limit *CI* = 0.006, 95% upper limit *CI* = 0.035, and on RT, *IE* = 0.153, 95% lower limit *CI* = −7.738, 95% upper limit *CI* = −3.230.

#### Experiment 2

A linear mixed model was conducted to explore the effect of music P/A (noise in the presence/absence of music) on subjective arousal. The model showed a significant main effect of music P/A, showing that subjective arousal was higher in the music-present condition than in the music-absent condition, *t*(1114.258) = −6.227, *p* < 0.001 (*b* = −0.200, *SE* = 0.032; see Fig. 4).

The linear mixed model exploring the relationship between subjective arousal and attentional-state category showed that, similarly to Experiment 1, task-focus states were linked to significantly higher arousal than mind-blanking states, *t*(4616) = −21.278, *p* < 0.001 (*b* = −1.263, *SE* = 0.059), than mind-wandering states, *t*(4616) = −40.439, *p* < 0.001 (*b* = −1.305, *SE* = 0.032) and than external-distraction states, *t*(4616) = −23.281, *p* < 0.001 (*b* = −0.818, *SE* = 0.035; see Fig. 4).

The mediation analyses showed that subjective arousal mediated the effect of music P/A (noise in the presence/absence of music) on the balance of mind-wandering versus task-focus states, *IE* = 0.367, 95% lower limit *CI* = 0.253, 95% upper limit *CI* = 0.489, on the balance of mind-wandering versus external-distraction states, *IE* = 0.173, 95% lower limit *CI* = 0.120, 95% upper limit *CI* = 0.233, and on RT, *IE* = −8.699, 95% lower limit *CI* = −11.964, 95% upper limit *CI* = −5.855.

### Serial mediation effects of mood valence and subjective arousal on the balance of mind-wandering versus task-focus states

Given that mood valence and subjective arousal separately mediated the effect of music P/A (relative silence in the presence/absence of music in Experiment 1, and noise in the presence/absence of music in Experiment 2) on mind-wandering and task-focus states, serial mediation analyses were performed focusing on the mediation effect of both mood valence and arousal together to see (i) whether there is a mediation where music P/A increases mood valence (mediator one) which increases subjective arousal (mediator two) which in turn leads to more frequent task-focus and less frequent mind-wandering states and (ii) whether there is a mediation where music P/A increases subjective arousal (mediator one) which increases mood valence (mediator two) which in turn leads to more frequent task-focus and less frequent mind-wandering states.

We used the 4.0 version of the PROCESS macro (an observed variable ordinary least squares and logistic regression path analysis modelling tool^91^) with bootstrapping procedures for the indirect effects (unstandardised indirect effects were computed for each of 5000 bootstrapped samples).

#### Experiment 1

The mediation analysis in which mood valence was mediator one and subjective arousal was mediator two showed that mood valence and subjective arousal serially mediated the effect of music P/A (relative silence in the presence/absence of music) on the balance of mind-wandering versus task-focus states, as predicted, *IE* = 0.229, 95% lower limit *CI* = 0.199, 95% upper limit *CI* = 0.262.

Similarly, the mediation analysis in which subjective arousal was mediator one and mood valence was mediator two showed that mood valence and subjective arousal serially mediated the effect of music P/A (relative silence in the presence/absence of music) on the balance of mind-wandering versus task-focus states, *IE* = −0.029, 95% lower limit *CI* = −0.043, 95% upper limit *CI* = −0.016.

#### Experiment 2

The mediation analysis in which mood valence was mediator one and subjective arousal was mediator two showed that, as predicted mood valence and subjective arousal serially mediated the effect of music P/A (noise in the presence/absence of music) on the balance of mind-wandering versus task-focus states, *IE* = 0.429, 95% lower limit *CI* = 0.358, 95% upper limit *CI* = 0.513.

However, the mediation analysis in which subjective arousal was mediator one and mood valence was mediator two did not show a significant serial mediation effect.

### Serial mediation effects of mood valence and subjective arousal on the balance of mind-wandering versus external-distraction states

Given that mood valence and subjective arousal separately mediated the effect of music P/A (relative silence in the presence/absence of music in Experiment 1, and noise in the presence/absence of music in Experiment 2) on mind-wandering and external-distraction states, two serial mediation analyses were performed to explore (i) whether there is a mediation where music P/A (relative silence in the presence/absence of music in Experiment 1, and noise in the presence/absence of music in Experiment 2) increases mood valence (mediator one) which increases subjective arousal (mediator two) which in turn leads to more frequent external-distraction and less frequent mind-wandering states and (ii) whether there is a mediation where music P/A increases subjective arousal (mediator one) which increases mood valence (mediator two) which in turn leads to more frequent external-distraction and less frequent mind-wandering states.

We used the 4.0 version of the PROCESS macro (an observed variable ordinary least squares and logistic regression path analysis modelling tool^91^) with bootstrapping procedures for the indirect effects (unstandardised indirect effects were computed for each of 5000 bootstrapped samples).

#### Experiment 1

The mediation analysis in which mood valence was mediator one and subjective arousal was mediator two showed that mood valence and subjective arousal serially mediated the effect of music P/A (relative silence in the presence/absence of music) on the balance of mind-wandering versus external-distraction states, *IE* = 0.041, 95% lower limit *CI* = 0.013, 95% upper limit *CI* = 0.070.

However, the mediation analysis in which subjective arousal was mediator one and mood valence was mediator two did not show a significant serial mediation effect.

#### Experiment 2

The mediation analysis in which mood valence was mediator one and subjective arousal was mediator two showed that mood valence and subjective arousal serially mediated the effect of music P/A (noise in the presence/absence of music) on the balance of mind-wandering versus external-distraction states, *IE* = 0.156, 95% lower limit *CI* = 0.120, 95% upper limit *CI* = 0.200.

However, the mediation analysis in which subjective arousal was mediator one and mood valence was mediator two did not show a significant serial mediation effect.

### Serial mediation effects of mood valence and subjective arousal on reaction time (RT)

Finally, given that mood valence and subjective arousal separately mediated the effect of music P/A (relative silence in the presence/absence of music in Experiment 1, and noise in the presence/absence of music in Experiment 2) on RT, two serial mediation analyses were performed to explore (i) whether there is a mediation where music P/A increases mood valence (mediator one) which increases subjective arousal (mediator two) which in turn leads to faster RT with the music and (ii) whether there is a mediation where music P/A increases subjective arousal (mediator one) which increases mood valence (mediator two) which in turn leads to faster RT with the music.

We used the 4.0 version of the PROCESS macro (an observed variable ordinary least squares and logistic regression path analysis modelling tool^91^) with bootstrapping procedures for the indirect effects (unstandardised indirect effects were computed for each of 5000 bootstrapped samples).

#### Experiment 1

The mediation analysis in which mood valence was mediator one and subjective arousal was mediator two showed that mood valence and subjective arousal serially mediated the effect of music P/A (relative silence in the presence/absence of music) on RT, *IE* = −8.420, 95% lower limit *CI* = −11.467, 95% upper limit *CI* = −5.594.

However, the mediation analysis in which subjective arousal was mediator one and mood valence was mediator two did not show a significant serial mediation effect.

#### Experiment 2

The mediation analysis in which mood valence was mediator one and subjective arousal was mediator two showed that mood valence and subjective arousal serially mediated the effect of music P/A (noise in the presence/absence of music) on RT, *IE* = −9.738, 95% lower limit *CI* = −12.638, 95% upper limit *CI* = −7.049.

However, the mediation analysis in which subjective arousal was mediator one and mood valence was mediator two did not show a significant serial mediation effect.

## Discussion

Across two experiments, the present study aimed to explore how mood and arousal mediate the effect of preferred background music on attentional state and performance on a simple vigilance task. It also aimed to generalise recent laboratory findings^5^ to an environment where there are more distractions around participants. To explore whether participants report a different pattern of attentional state changes in their homes than in the laboratory, Experiment 1 was conducted against a backdrop of relative silence with presumably only infrequent distractions around participants, while Experiment 2 was conducted against a backdrop of continuous distractions in the form of experimentally controlled office noise. Before discussing results related to the main focus of the study on the mediation effect of mood and arousal, we discuss the results of baseline analyses that aimed to replicate past findings and that provided important groundwork for the mediation analyses.

Focusing on the effect of background music on attentional state, in both experiments, background music decreased mind-wandering states and increased task-focus and external-distraction states. The decrease in mind-wandering and increase in task-focus states with the music is consistent with the laboratory version of the current study^5^ and with a replication and extension of this laboratory version looking at attentional state during a different vigilance task (a perceptual decision task including Gabor patches^33^). Interestingly, when music is listened to without a concurrent task, it has been found to increase rather than decrease mind-wandering (e.g.,^3,92–96^). Differences between findings when music is the primary activity as compared to when it is listened to in the background might be due to differences in people’s baseline arousal levels as well as their inclination to shift their focus of attention internally versus externally with the music: when music listening is the primary activity, people might turn to music to relax (see, e.g.,^97^) and induce the *internal* state of mind wandering^98–104^. On the other hand, when music listening is paired with an *external* task that is monotonous and boring—such as the vigilance task performed here—people might choose music for its energising and arousal-increasing effect (see, e.g.,^4^) and to help them maintain and/or shift their attentional focus externally which in-turn results in a decrease in internal mind-wandering states.

Consistent with the idea that music paired with a boring external task can shift the focus of attention externally, there was an increase in external-distraction states with music, along with the increase in externally oriented task-focus states. This is a new finding not previously shown in the laboratory study of Kiss and Linnell^5^ but consistent with the findings of Varao-Sousa et al.^64^ in a more natural environment and suggests that when there is at least some level of external distraction around participants, regardless of the level of this distraction, participants experience more external-distraction states with music. The similarity of the findings across Experiments 1 and 2 regardless of the level of distraction involved in these experiments might, however, have been a consequence of the manipulation of noise in Experiment 2: given that the noise included in this experiment was played continuously throughout the entire experiment and was relatively predictable, it might be that participants habituated to it rather than experiencing additional distractions in its presence. This possibility is further supported by the fact that participants reported similar levels of sudden, infrequent distractions around them in both experiments (regardless of the extra noise in Experiment 2).

As well as background music increasing task-focus states in both experiments, it was linked to faster reaction times in both experiments, and a decrease in false alarms in Experiment 2. This positive effect of music on vigilance task-performance extends the findings of the study by Kiss and Linnell^5^ which found no effect of background music on reaction time and is in keeping with the findings of the study by Homann et al.^33^ which showed decreased reaction time variability with background music. It is also in keeping with the findings of other studies showing better performance with background music on a variety of vigilance tasks (including, e.g., a visual vigilance task where participants had to detect changes in the brightness of a light^105^, a signal detection task^106^, simulated driving^28,107^, and a computer motor racing game^16,108^.

In addition to the link between reaction time and music, reaction-time performance in both experiments was linked to attentional state, similarly to in both Kiss and Linnell^5^ and Unsworth and Robison^13^. Specifically, task-focus states were linked to faster reaction times than mind-wandering or external-distraction states. This linkage between attentional state and performance suggests that the subjective attentional state measure used here (subjectively reported thought-probe responses collected throughout the task) is a valid measure of fluctuations in attentional state as expressed in behaviour.

Further examining attentional state and its link now with subjective arousal showed that, as expected, in both experiments there was a significant link between subjective arousal and attentional state. Nonetheless, the direction of this link was only partially consistent with Unsworth and Robison’s^13^ findings on attentional state and *objectively* measured arousal. Specifically, the present results showed that task-focus states were linked to the highest arousal level, followed by external-distraction and mind-wandering states. The fact that external-distraction states were linked to lower subjective arousal than task-focus states is not consistent with Unsworth and Robison’s^13^ pupillometric findings showing that external-distraction states were linked to higher arousal than task-focus states. Nonetheless, the present results make sense according to the model developed by Lenartowicz et al.^47^, as in this model it is hypothesised that external-distraction states are distinguished from mind-wandering states not by arousal levels but by an external rather than internal focus of attention, and that both external-distraction and mind-wandering can be associated with sub-optimal and supra-optimal arousal levels. One difference between Unsworth and Robison^13^ and the present study is that the present study involved only participants who choose to listen to background music: assuming that background music is used by people whose baseline arousal is lower, we might have been testing a less aroused population than that of Unsworth and Robison^13^. In fact, in keeping with external-distraction states being linked to sub-optimal arousal here, recent results of our pupillometric study (Kiss, Szikora, & Linnell^61^)—that had the same inclusion criteria regarding background music listening—also found external-distraction states to be linked to lower arousal than task-focus states. In this context, the effects of background music reported here may indicate that background music is not always arousing enough to support an increase in task-focus states, but still shifts the focus of attention externally and so in these cases results in low-arousal external-distraction states instead of task-focus states.

In addition to the link between subjective arousal and attentional state, there was also a link between mood valence and attentional state, such that in both experiments task-focus states were linked to more positive mood than off-task external-distraction and mind-wandering states. These findings are compatible with studies showing that mind-wandering decreases mood valence (e.g.,^6–12^).

Analysing the role of mood and arousal further, we directly tested the suggestion made in Kiss and Linnell^5^ that music increases arousal and extended the test to examine if music also increases mood. Results showed that background music indeed increased both positive mood and subjective arousal in both experiments. These results make sense considering that music decreased mind-wandering and increased task-focus and external-distraction states, and that mind-wandering was linked to the lowest mood and arousal levels out of the three attentional states. Music increasing arousal as well as mood in both experiments is supported by past studies showing an increase in mood (e.g.,^62^) and arousal (e.g.,^15,16^) with background music and especially with preferred and liked music^2^. It is also consistent with research suggesting that during simple tasks people mostly listen to music for enjoyment and energising^4,109^, highlighting the potential importance of mood and arousal in explaining the effects of background music.

Importantly, mood and arousal—separately and together—mediated the effect of background music on the balance of mind-wandering versus task-focus states, and the balance of mind-wandering versus external-distraction states, in both experiments. Additionally, mood and arousal mediated the effect of background music on reaction time. Compatible with both mood and arousal mediating performance, the mood-and-arousal hypothesis predicts that the increase in *performance* with music is the result of increased mood and increased arousal^58–60^, although this hypothesis refers to music listening *prior to* task-performance. Studies extending the role of mood and arousal to specifically background music listening have shown mixed results (e.g.,^6,81, 110–114^). The present findings highlight the relevance of the mood-and-arousal hypothesis in the context of background music listening during performance of a simple vigilance task and extend previous work on performance to attentional state (by showing decreases in mind-wandering and increases in task-focus and external-distraction states). Importantly, the serial mediation effects of mood and arousal were mostly confined to models where mood was the first mediator and arousal the second. When arousal was the first mediator (and mood the second), the serial mediation effects were not significant in most cases. This suggests that when considering how mood and arousal may together mediate the effect of background music on attentional state and reaction time performance, the effects of mood precede those of arousal.

With regard to limitations of the current study, in this study only people who normally listen to music during attention-demanding tasks were included. Although this inclusion criterion increased ecological validity—as it allowed participants to choose music tracks for the study that they would normally listen to—restricting the participation only to them presumably meant that all participants liked listening to background music and moreover were used to listening to it while performing a concurrent cognitively demanding task. This in-turn could have increased the possibility of finding a positive effect of the music in this study. Thus, the present results can only generalise to those individuals who use background music. In the future, studies could involve a broader participant pool and compare those who prefer versus do not prefer background music listening.

In summary, the current results show that preferred background music—presented against backdrops of both relative silence and continuous noise—decreased mind-wandering states and increased task-focus and external-distraction states, together with positive mood and subjective arousal. It also affected objectively measured performance on the Psychomotor Vigilance Task and led to faster reaction times (and less false alarms in Experiment 2). Importantly, results also showed for the first time that positive mood and subjective arousal, both separately and together, mediated the effect of music on attentional state (the balance of mind-wandering versus task-focus states, and mind-wandering versus external-distraction states), in addition to mediating the effect of music on performance as indexed by reaction time. Also importantly, the present results provide support for and shed light on the mood-and-arousal hypothesis by suggesting that, where the effects of background music are mediated by both mood and arousal, the effects of mood precede those of arousal. Overall, these results highlight that unlike in scenarios where music listening is the primary activity and where it has been shown to relax people and increase internally oriented mind-wandering states, in scenarios where music is listened to during the performance of a boring vigilance task, it decreases these mind-wandering states—and increases externally oriented task-focus states and external-distraction states—by increasing mood and arousal.

## Data Availability

The datasets obtained during, and/or analysed during, the current study are available on the Open Science Framework via https://osf.io/8u6bm/?view_only=6cb767fe69ea484982c5ed578423529e (10.17605/OSF.IO/8U6BM).
