# Author Correction: The role of mood and arousal in the effect of background music on attentional state and performance during a sustained attention task

**DOI:** 10.1038/s41598-024-61470-z

**Published:** 2024-05-13

**Authors:** Luca Kiss, Karina J. Linnell

**Affiliations:** Department of Psychology, Goldsmiths University of London, 8 Lewisham Way, New Cross, London, SE14 6NW UK

Correction to: *Scientific Reports* 10.1038/s41598-024-60218-z, published online 25 April 2024

The original version of this Article contained errors.

In the original version of this Article, the URL in Reference 77 was incorrect.

Reference 77

“Spotify AB. Spotify for Developers (2018). https://developer.spoti fy.com. Retrieved 10 Jan 2019.”

now reads,

“Spotify AB. Spotify for Developers (2018). https://developer.spotify.com. Retrieved 10 Jan 2019.”

In addition, the Acknowledgements section was incomplete. It now reads,

“The authors would like to thank Bence Szikora for the coding of the online version of the Psychomotor Vigilance Task used here and for his invaluable technical expertise and advice throughout the completion of this study. We also thank one anonymous reviewer and Steffen Herff for their helpful comments on the manuscript.”

The original Article has been corrected.

